# Use of Pearson’s Chi-Square for Testing Equality of Percentile Profiles across Multiple Populations

**DOI:** 10.4236/ojs.2015.55043

**Published:** 2015-08

**Authors:** William D. Johnson, Robbie A. Beyl, Jeffrey H. Burton, Callie M. Johnson, Jacob E. Romer, Lei Zhang

**Affiliations:** 1 Department of Biostatistics, Pennington Biomedical Research Center, Louisiana State University, Baton Rouge, USA; 2 Office of Health Data and Research, Mississippi State Department of Health, Jackson, USA

**Keywords:** Asymptotic Chi-Square Test, Equality of Percentiles, Large Sample Test, Median Test, Nonparametric Methods

## Abstract

In large sample studies where distributions may be skewed and not readily transformed to symmetry, it may be of greater interest to compare different distributions in terms of percentiles rather than means. For example, it may be more informative to compare two or more populations with respect to their within population distributions by testing the hypothesis that their corresponding respective 10^th^, 50^th^, and 90^th^ percentiles are equal. As a generalization of the median test, the proposed test statistic is asymptotically distributed as Chi-square with degrees of freedom dependent upon the number of percentiles tested and constraints of the null hypothesis. Results from simulation studies are used to validate the nominal 0.05 significance level under the null hypothesis, and asymptotic power properties that are suitable for testing equality of percentile profiles against selected profile discrepancies for a variety of underlying distributions. A pragmatic example is provided to illustrate the comparison of the percentile profiles for four body mass index distributions.

## 1. Introduction

Student’s t-test and analysis of variance are frequently employed to test the hypothesis that two or more distributions have common means. However, many random variables may have skewed distributions that are not readily transformed to symmetry, rendering the distributional assumptions that underlie use of these methods inappropriately. Nonparametric procedures such as the Wilcoxon, Kolmogorov-Smirnov and median tests are desirable alternatives to test for differences in distributions [1]. Unfortunately, many nonparametric tests are “global” tests of equivalence—that is, tests of whether the distributions are identical over the entire domain. For example, if the t-test is used to compare two distributions that are symmetrical and similarly shaped except for possible shifts in location, it may outperform the median test. However, if the sample sizes are small and the distributions are highly skewed the median test may be preferred. These tests are not designed to pin-point where the distributions are unequal or simultaneously test for differences in more than one distribution parameter. Similarly, under appropriate assumptions the t-test and variance-test are especially powerful for detecting differences in the location and scale, respectively, but may be considered too narrow in scope as they both test only one parameter. In practice, it is not uncommon to compare two distributions with varying degrees of skewness, location shifts, and possibly even mixtures of distributions. In these circumstances it may be of greater interest to compare the distributions in terms of their percentiles rather than their means or an overall test of equivalence. For example, it may be more informative to compare two or more distributions by testing the hypothesis that their profiles of judiciously selected percentiles are equal, where a percentile profile is defined as a set of one or more percentiles.

The procedure investigated here, first described by [2], can be thought of as a generalization of the median test. Instead of testing the equality of only one percentile—the 50^th^—the method is extended to simultaneously test multiple percentiles. In this way, it is possible to test if two or more sets (profiles) of desired percentiles are jointly identical across multiple populations. As an application of Pearson’s chi-square test, this approach has excellent large sample properties. We give an example of the procedure to compare several populations with respect their percentile profiles using body mass index (BMI) data from the National Health and Nutrition Examination Survey (NHANES).

We begin in Section 2 with a general formulation of hypotheses for comparing percentile profiles coupled with a testing strategy that is a novel generalization of that employed in the median test. Empirical power simulation results are shown in Section 3 to illustrate the test’s large sample properties under selected conditions with irregularly shaped distributions. An illustrative example applied to the NHANES data is presented in Section 4. Some concluding remarks on the test and planned future work are given in Section 5.

## 2. Formulation of Hypothesis and Test Procedure

Let Y denote a continuous random variable of interest and let *Q*_1_, *Q*_2_, ···, *Q_p_* denote a set of *p* percentiles (quantiles) that in some sense characterize the distribution of the random variable across its range. Further let *y*_1_, *y*_2_, ···, *y_n_* represent a random sample of observations and let *q*_1_, *q*_2_, ···, *q_p_* represent, respectively, the usual sample estimates of *Q*_1_, *Q*_2_, ···, *Q_p_*. Suppose random samples are available from each of *K* populations with percentiles *Q_h_* = *Q_h_*_1_, *Q_h_*_2_, ···, *Q_hp_*, *h* = 1, 2, ···, *K* where there is interest in testing the hypothesis that the percentile profiles are identical across the *K* populations; that is, interest is in testing:

$$H_{0}:Q_{1}=Q_{2}=\cdots =Q_{K}$$

The following approach is an extension of the median test:

1. Combine the *K* samples and obtain the usual estimates of the population percentiles for the corresponding combined populations.
2. Let *y* denote an arbitrary observation in the combined sample. Use the combined sample percentile estimates to define *p* categories or bins denoted bin_1_,bin_2_, ···,bin*_p_*_+1_, where bin_1_ = min(*y*) ≤ all *y* ≤ *q*_1_, bin_2_ = *q*_1_ < all *y* ≤ *q*_2_, ···, bin*_p_*_+1_ = *q_p_* < all *y* ≤ max where min and max, respectively, represent the minimum and maximum observations in the sample.
3. Construct a *K* by (*p* + 1) dimensional contingency table where the *h*^th^ row (*h* = 1, 2, ···, *K*) of the table is obtained by determining separately for each sample the number and proportion of observations in each bin (column) defined in step 2. Note that, the percentile profiles are identical across the *K* populations if and only if the underlying population proportions are identical within each column of the table.
4. Use Pearson’s chi-square statistic to test for homogeneity of the profiles of row percentages.

## 3. Power Simulations

**Figure 1:**
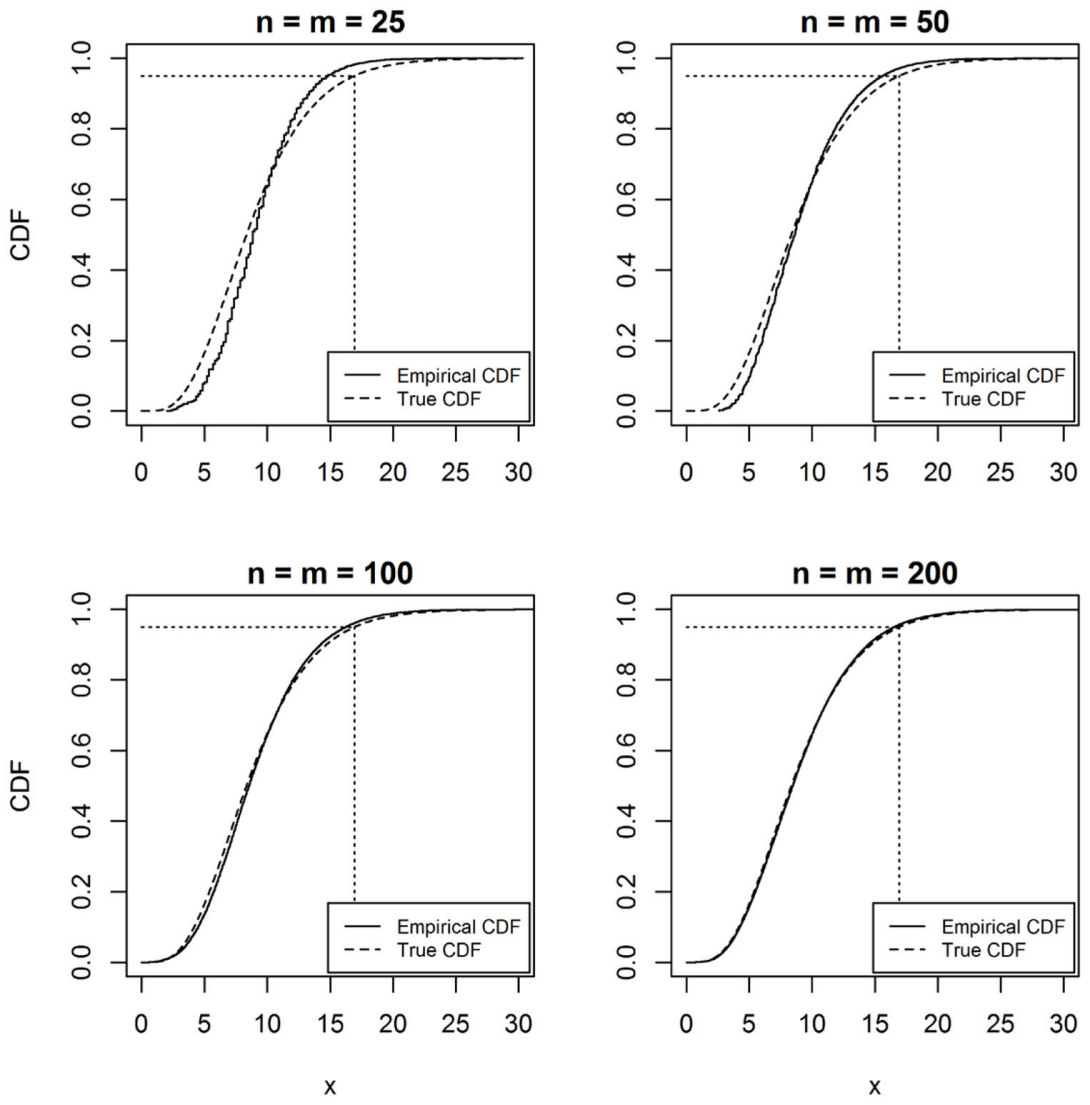
True chi-squared Cumulative Distribution Function (CDF) compared to empirical CDF of test statistic from 100,000 replicate samples under *H*_0_: *Q*_1_ = *Q*_2_ where *Q* = (0.01, 0.05, 0.1, 0.25, 0.5, 0.75, 0.9, 0.95, 0.99) based on simulated samples from the Gamma (shape = 2, scale = 3) distribution.

The asymptotic properties of the proposed percentile test were investigated. For example, Figure 1 shows the convergence of the distribution of the test statistic to a true chi-squared distribution with nine degrees of freedom when comparing the percentile profiles (1^st^, 5^th^, 10^th^, 25^th^, 50^th^, 75^th^, 90^th^, 95^th^, and 99^th^) of two populations with sample size *n* and *m* from the Gamma (shape = 2, scale = 3) distribution for simulated samples of various sizes (*n* = *m* = 25, 50, 100, 200). There is good agreement between empirical and true chi-squared distributions at around sample size 100, becoming nearly indistinguishable by samples of size 200. Simulations show that increasing the number of percentiles increases the sample size required for convergence to the true chi-squared distribution. The example in Figure 1 could be considered a relatively extreme case in that it simultaneously tests nine percentiles, many of which are at the extreme tails of the distribution. Profiles with fewer percentiles (and closer to center of the distribution) converge satisfactorily with smaller sample sizes.

Power simulations were conducted for various scenarios where the data were generated from the following family of distributions: (1) gamma, (2) mixtures of gammas, and (3) uniform. Symmetric distributions were not considered as better procedures exist for their comparisons. The motivation for this procedure is to compare the profiles of asymmetric distributions that are common in biostatistics applications; gamma distributions are a natural choice to simulate skewed distributions due to their flexibility in simulating data from irregularly shaped distributions with wide-ranging shift options. The properties of the test when using a single percentile were also investigated. The percentile test is applicable to a wide variety of distributions, but is especially useful when comparing skewed and/or multimodal data and detecting differences in ranges between uniform distributions. All power estimates are based on 100,000 replicate samples and all procedures were programmed and carried out with R 3.1.2.

### 3.1. Gamma Distributions

Results of simulation studies to examine properties of the percentile test for comparing gamma distributions are presented in Table 1 and Table 2. The empirical alpha estimates under *H*_0_ can be found in Table 1 for testing random samples from equivalent gamma distributions while the power estimates under *H*_1_ for testing random samples from two unequal gamma distributions are in Table 2. For estimating power, data were generated for the two populations from gamma distributions differing in both scale and shape parameters (see Table 2 for details). Several percentile profiles were tested for each scenario: *P*_1_, *P*_3_, *P*_5_, *P*_7_, and *P*_9_. These refer to tests of the percentile profiles (0.5), (0.25, 0.5, 0.75), (0.1, 0.25, 0.5, 0.75, 0.9), (0.05, 0.1, 0.25, 0.5, 0.75, 0.9, 0.95), and (0.01, 0.05, 0.1, 0.25, 0.5, 0.75, 0.9, 0.95, 0.99), respectively. These profiles were used for the remaining simulations in the paper.

The empirical 95^th^ percentile of the 100,000 replicate samples—the empirical alpha—converges to the true 95^th^ percentile for each profile tested and its respective chi-squared distribution as the sample size increases. Not surprisingly, the empirical alpha does not match the true chi-squared 95^th^ percentile for small sample sizes but is very close with sample sizes as small as 100 for some profiles. The test for the *P*_1_ profile, which is equivalent to the median test, approaches 0.05 from above while profiles with more than one percentile approach 0.05 from below with longer profiles converging slower and starting closer to zero. This is due to the increase in the number of bins in the contingency table and hence the degrees of freedom in the chi-square test; and, as will be shown later, smaller expected values in cells result in smaller chi-square values while holding row profiles equal.

**Figure 2:**
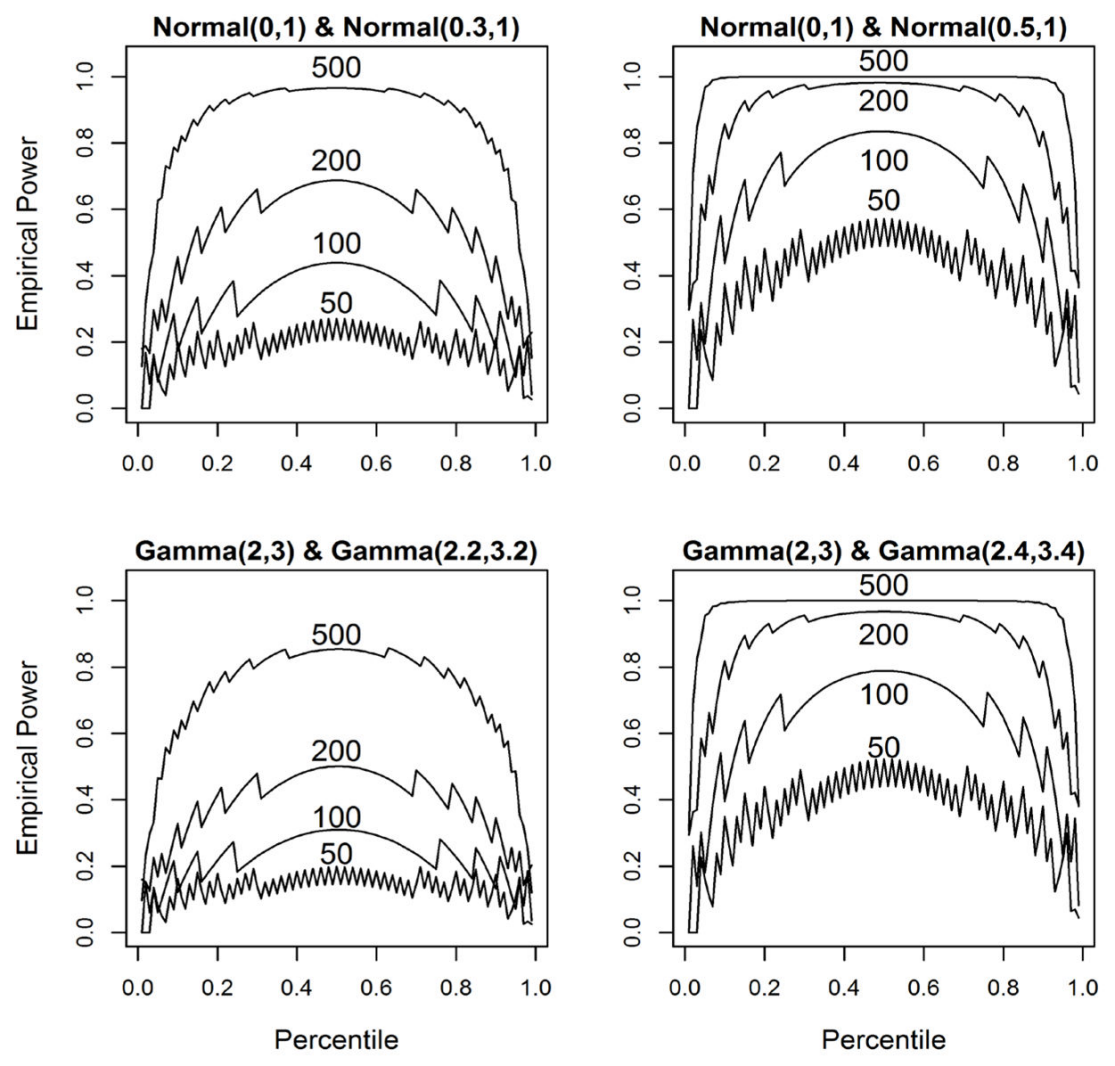
Power Simulations of testing a single percentile from 0.01 to 0.99 in 0.01 increments for normal and gamma distributions for n = 50, 100, 200, 500.

Table 2 shows the differences in the empirical power estimates for various percentile profiles, *P*_1_ through *P*_9_. For the first example (Gamma (shape = 2.2, scale = 3.2)), the basic median test, *P*_1_, is the most powerful for all sample sizes. Because the test’s (and the chi-squared test’s) power is a function of the true difference of percentiles between the distributions and the sample size, the median test performs the best (see Figure 2 comparing distributions with constant difference in percentiles). When testing profile *P*_9_, for instance, the count in the final bin is just 5 for each group with balanced samples of size 500 which has limited contribution to the overall chi-squared with so many degrees of freedom.

This is also the case in the second example (Gamma (shape = 2.4, scale = 3.4)) in Table 2. The median test again is the most powerful. However, one must keep in mind that these particular percentile profiles were more or less arbitrarily chosen and used throughout the paper for consistency. While these choices seem appropriate for symmetric distributions, other choices may be preferred for gamma as well as for other asymmetric distributions. Thus, in practical applications, the analyst would likely select percentiles that are appropriate for the specific data at hand. For the first example, if a profile of (0.5, 0.75, 0.9, 0.95) is tested, the power estimate increases to 1.000 for sample size of 200, compared to the 0.503 for *P*_1_.

### 3.2. Mixture of Gamma Distributions

The convergence to 0.05 is nearly identical to the single gamma case for each combination of sample size and percentile profile tested. This result shows the test statistic converges to chi-squared for a wide range of distributions (results were consistent for simulations with normal and uniform distributions, although they are not shown).

The empirical power estimates for comparing mixtures of gammas are presented in Table 3. Similar to the previously described simulation study, the distributions used for generating data for the two populations differed in both shape and scale parameters. In this case, these parameters differed between the populations in both of the gammas making up the mixture distributions. As expected, the power increases as sample size increases with the power greater than 0.9 at around sample sizes of 500. In these mixtures of gammas examples, the median test (*P*_1_) generally performs the worst of all profiles, unlike in the example using the single gamma. For sample sizes greater than or equal to 100, *P*_1_ is the least powerful of all the profiles tested and only better than *P*_9_ for sample size 50. For sample size 25, *P*_1_ is the most powerful due to the properties of the chi-square test, *i.e*. insufficient observations in the contingency table for profiles with more than one percentile.

### 3.3. Uniform Distributions

Simulation-based empirical power estimates for comparing uniform distributions are shown in Table 4 and a comparison with estimated power from other procedures is presented in Table 5. Simulations (not shown) confirmed the asymptotic behavior of testing uniform distributions under *H*_0_. The empirical alpha for each sample size/profile combination was equivalent (within 0.01) for uniform distributions as for gammas and mixtures of gammas. We considered two scenarios: (1) a shift in the range of the distribution from uniform (0, 1) to (0.1, 1.1) and (2) and reduction in the range from uniform (0, 1) to (0.1, 0.9). Table 4 shows the results of testing the percentile profiles between sample data from the uniform (0, 1) and these two modified uniform distributions. The percentile profiles *P*_1_, *P*_3_, *P*_5_, *P*_7_, and *P*_9_ are the same as those previously used for testing the gamma distributions. The power estimates from the percentile test (P) used in Table 5 are specifically chosen for testing uniform distributions.

The percentiles chosen in simulations in Table 5 are based on the properties of the uniform distribution. When comparing uniform distributions, the differences can be detected at the extreme percentiles, near 0 and 1— the middle part of either distribution is unnecessary. For instance, if the lower boundaries for the uniform distributions are unequal, a percentile near 0 will detect the difference (there is always a percentile as a function of the sample size that will create a perfect separation of observations into the first bin). Similarly, if the upper boundaries are unequal, there always exists a percentile near 1 that perfectly separates the observations. This results in a large chi-squared statistic and the rejection of the null hypothesis. If sample sizes are balanced for each group, a good choice of percentiles for comparing uniform distributions is (1/*n* + δ, 1 − (1/*n* + δ)) where *n* is the sample size of one group and δ is a small added value (add a 1 in the furthest decimal place, *i.e*. 0.03 would become 0.031 and 0.005 would become 0.0051, etc.). In the example, for samples of size 100, the optimal percentiles would be (0.011, 0.989). We will refer to the sample size dependent percentiles for uniform distributions as uniform optimal percentiles (UOPs).

The percentile test is extremely powerful in detecting differences in the range of uniform distributions for both scenarios: (1) a shift (with equal range) and (2) a change in range but with equal average value. The profiles *P*_1_, *P*_3_, *P*_5_, *P*_7_, and *P*_9_ displayed a range of performance with *P*_7_ having the highest power for both scenarios. Although the *P*_7_ profile performs well, the power is greatly improved when UOPs are used. When these sample size dependent percentiles are used, power of greater than 0.8 is achieved with samples of less than 50 in testing scenario (1) and (2). The performance is substantially better than both Wilcoxon and Kolmogorov-Smirnov tests, particularly under scenario (2), a change in range but with equal expected value. For example, the power of the percentile test with UOPs at sample size of 50 is 0.868, compared to 0.051 and 0.072 for the Wilcoxon and Kolmogorov-Smirnov tests, respectively.

### 3.4. Testing Profiles of 100 Percentiles

Additional simulations were conducted to examine the behavior of tests that compare profiles each comprised of 100 percentiles. Since the power of the percentile profile test is a function of sample size and the true difference in the distributions with respect to their percentile profiles, normal distributions were used to eliminate one of these variables (the difference in all percentiles are equal to the difference in the location parameter, assuming the scale parameter is constant). As can be seen in the plots in Figure 2, the median test (0.5 percentile) is the most powerful single-percentile test for each sample size even though the differences in percentiles between distributions are the same. This is likely due to the nature of the chi-squared test—as the differences in observed and expected values increases linearly, the chi-squared value increases quadratically. However, this relationship holds for gamma distributions for which the difference between percentiles is not constant. When comparing gamma (shape = 2, scale = 3) and gamma (shape = 2.2, scale = 3.2), the difference in the true percentiles increases as the percentile increases from zero to one.

For small sample sizes, the power fluctuates greatly as the percentile changes and exhibits a pronounced “saw tooth” behavior. However, these fluctuations gradually disappear as the sample size increases. As the sample size increases, the power increases as the percentile is held constant. The same is true of the difference in the distributions’ percentiles. The power is generally symmetric with a maximum at percentile 0.5 for all cases even if the differences in the percentiles between the distributions are not constant. The relationship between the true difference in distributions, sample size and percentile is quite complex and needs to be investigated further to be fully understood. Understanding these relationships will likely improve the effectiveness of this procedure.

## 4. Illustrative Example

**Figure 3:**
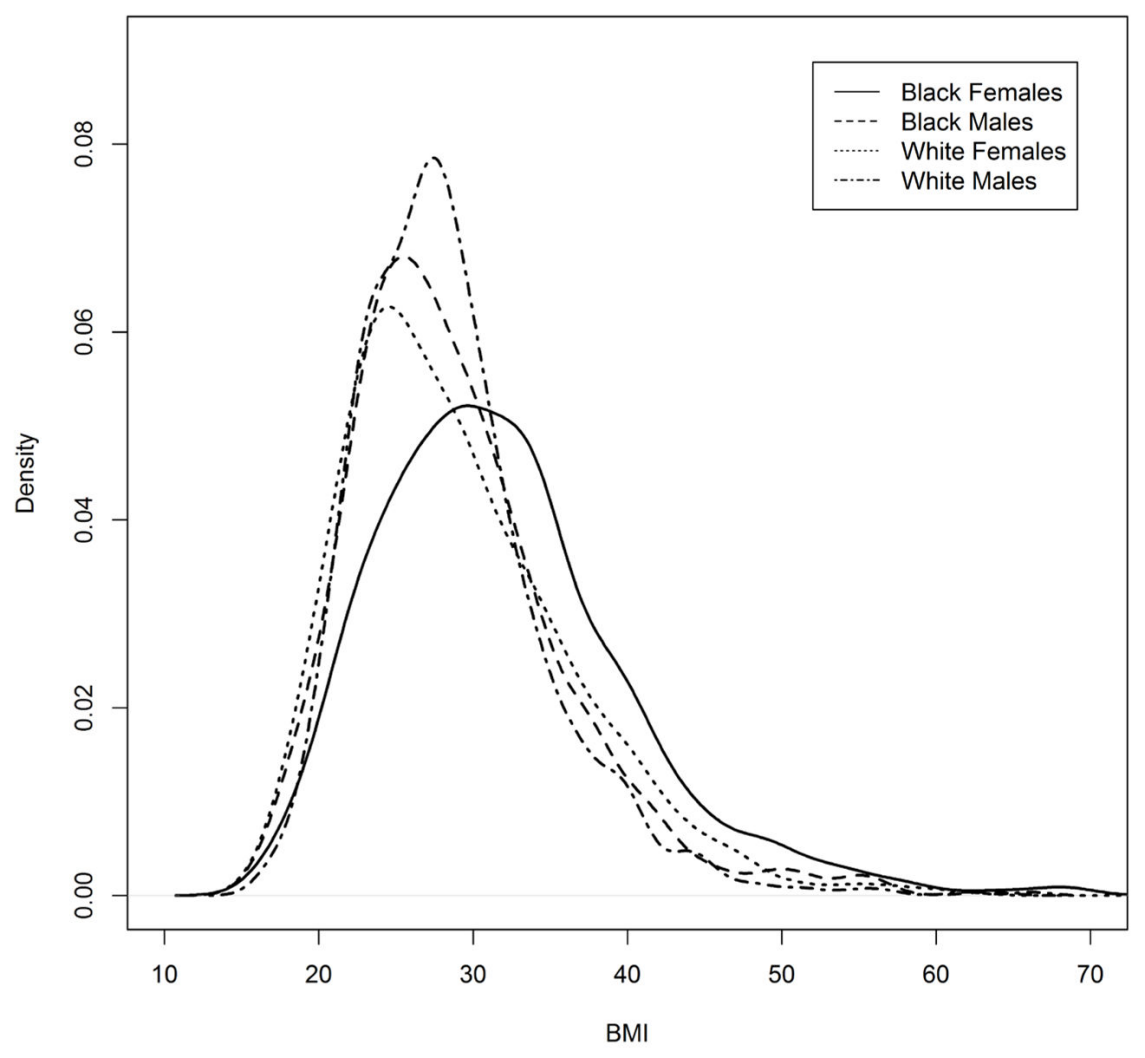
Kernel density estimates of BMI for adult black females, black males, white females, and white males between ages 20 and 79.

Body mass index (BMI) data from the 2011–2012 NHANES study were used as an example of an application of the percentile test. For illustrative purposes, only non-Hispanic black and white adults between the ages of 20 and 79 are included in the analysis. Suppose there was interest in testing the homogeneity of BMI percentile profiles for independent race-sex groups: black females, black males, white females, and white males. Observed discrepancies among the four BMI distributions are shown in Figure 3. To test for homogeneity of the profiles, one could follow the steps outlined in Section 2. Consider the sample obtained by combining the four race-sex groups, and its 1^st^, 5^th^, 10^th^, 25^th^, 50^th^, 75^th^, 90^th^, and 99^th^ BMI percentiles, shown in Table 6.

To illustrate, consider the two percentile sets (0.25, 0.5, 0.75) and (0.1, 0.25, 0.5, 0.75, 0.9) where interest is in testing homogeneity of each of the corresponding percentile profiles. The corresponding percentile profiles obtained from Table 6, (24.5, 28.5, 33.5) and (22.0, 24.5, 28.5, 33.5, 39.2), were used as sets of cutoff values to construct the contingency tables in Table 7 and Table 8, respectively. Applying the chi-square test using the percentile set (0.25, 0.5, 0.75) in Table 7 results in a highly significant difference between the percentile profiles (p < 0.0001). Similarly, the profile (0.1, 0.25, 0.5, 0.75, 0.9) in Table 8 is also highly significantly different (p < 0.0001). To further test differences between the group profiles, within gender pairwise comparisons between black males and white males as well as black females and white females were performed. No significant difference was found between white males and black males for either set of percentiles (p = 0.192 and p = 0.298 for (0.25, 0.5, 0.75) and (0.1, 0.25, 0.5, 0.75, 0.9), respectively). However, black females differed significantly from white females in both sets of percentile profiles (p < 0.0001 for both sets).

## 5. Concluding Remarks

Percentile profiles provide easy to interpret characterizations of data distributions and are frequently used as descriptive statistics to capture distributional variations other than shifts in central location. Although the median test is well known, methods of conducting simultaneous inferences about percentiles within a specified profile have not been well described. The approach used in this manuscript is based on well-known fundamental principles that are easy to understand and implement. One clear advantage of this procedure over other tests is the ability to directly compare a number of percentiles between distributions rather than overall tests of equality or changes in location or shape.

The procedure is extremely powerful in detecting differences between uniform distributions. When percentiles are optimally chosen, the power of the percentile test outperforms other procedures and is well powered at relatively small sample sizes. Further work will be done to investigate the properties of the test in comparing uniform distributions.

A limitation is that the test relies on large sample theory and further study is needed to evaluate the severity of this restriction. It is important to remember that there are more powerful tests for comparing overall equality of distributions (Wilcoxon, KS test) or differences in specific parameters (t-test, F-test), but none that test equality of a set of multiple percentiles between distributions. Rules for choosing percentiles to maximize power may be useful research.

## Figures and Tables

**Table 1:** Empirical alpha estimates for comparing Gamma distribution.

*n = m*	Gamma (shape = 2, scale = 3)				
	*P*_1_	*P*_3_	*P*_5_	*P*_7_	*P*_9_
25	0.088	0.047	0.044	0.031	0.017
50	0.072	0.056	0.048	0.043	0.028
100	0.066	0.053	0.049	0.047	0.038
200	0.059	0.050	0.049	0.048	0.043
500	0.050	0.049	0.050	0.050	0.048

**Table 2:** Empirical power estimates when testing against Gamma (shape = 2, scale = 3).

*n = m*	Gamma (shape = 2.2, scale = 3.2)					Gamma (shape = 2.4, scale = 3.4)				

	*P*_1_	*P*_3_	*P*_5_	*P*_7_	*P*_9_	*P*_1_	*P*_3_	*P*_5_	*P*_7_	*P*_9_
25	0.162	0.081	0.068	0.045	0.024	0.356	0.200	0.154	0.097	0.051
50	0.200	0.143	0.108	0.087	0.057	0.524	0.430	0.348	0.276	0.187
100	0.311	0.235	0.193	0.165	0.127	0.789	0.740	0.682	0.616	0.530
200	0.503	0.437	0.390	0.334	0.286	0.966	0.967	0.959	0.941	0.915
500	0.854	0.851	0.827	0.782	0.742	1.000	1.000	1.000	1.000	1.000

**Table 3:** Empirical power estimates testing against 1/2 Gamma (shape = 1.5, scale = 2.5 & 1/2 Gamma (shape = 4.5, scale = 4.5).

*n = m*	1/2 Gamma (shape = 1.8, scale = 2.7) & 1/2 Gamma (shape = 4.3, scale = 6)					1/2 Gamma (shape = 1.8, scale = 2.2) & 1/2 Gamma (shape = 3, scale = 8)				

	*P*_1_	*P*_3_	*P*_5_	*P*_7_	*P*_9_	*P*_1_	*P*_3_	*P*_5_	*P*_7_	*P*_9_
25	0.117	0.092	0.086	0.053	0.027	0.091	0.065	0.069	0.048	0.024
50	0.113	0.176	0.171	0.135	0.076	0.071	0.099	0.119	0.103	0.058
100	0.142	0.309	0.357	0.318	0.236	0.067	0.145	0.223	0.221	0.160
200	0.194	0.569	0.690	0.662	0.597	0.059	0.244	0.457	0.475	0.417
500	0.358	0.946	0.988	0.988	0.984	0.056	0.559	0.893	0.922	0.924

**Table 4:** Empirical power estimates when testing against Uniform (0, 1).

*n = m*	Uniform (0.1, 1.1)					Uniform (0.1, 0.9)				

	*P*_1_	*P*_3_	*P*_5_	*P*_7_	*P*_9_	*P*_1_	*P*_3_	*P*_5_	*P*_7_	*P*_9_
25	0.170	0.107	0.154	0.097	0.031	0.090	0.089	0.162	0.120	0.036
50	0.217	0.211	0.383	0.432	0.215	0.073	0.150	0.382	0.508	0.275
100	0.339	0.370	0.696	0.929	0.856	0.067	0.243	0.686	0.942	0.889
200	0.545	0.661	0.955	0.999	0.999	0.059	0.443	0.948	0.999	0.999
500	0.889	0.976	1.000	1.000	1.000	0.051	0.855	1.000	1.000	1.000

**Table 5:** Empirical power estimates when testing against Uniform (0, 1) with uniform percentile rule (P), Wilcoxon test and Kolmogorov-Smirnov (KS) test.

*n = m*	Uniform (0.1, 1.1)			Uniform (0.1, 0.9)		

	P	Wilcoxon	KS	P	Wilcoxon	KS
25	0.450	0.208	0.103	0.394	0.053	0.049
50	0.881	0.377	0.199	0.868	0.051	0.072
100	0.998	0.647	0.367	0.998	0.051	0.124
200	1.000	0.914	0.725	1.000	0.053	0.388
500	1.000	1.000	1.000	1.000	0.051	0.998

**Table 6:** Percentiles for black females, black males, white females, white males combined.

Percentile	0.01	0.05	0.1	0.25	0.5	0.75	0.9	0.95	0.99
Percentile Value	17.9	20.4	22.0	24.5	28.5	33.5	39.2	43.1	54.2

**Table 7:** Contingency table used with cutoffs corresponding to percentiles (0.25, 0.5, 0.75).

Group	Bin			
	1 (≤24.5)	2 (>24.5, ≤28.5)	3 (>28.5, ≤33.5)	4 (>33.5)
Black Females	108	140	186	259
Black Males	178	176	159	133
White Females	257	190	190	209
White Males	219	266	224	149

**Table 8:** Contingency table used with cutoffs corresponding to percentiles (0.1, 0.25, 0.5, 0.75, 0.9).

Group	Bin					
	1 (≤22.0)	2 (>22.0, ≤24.5)	3 (>24.5, ≤28.5)	4 (>28.5, ≤33.5)	5 (>33.5, ≤39.2)	6 (>39.2)
Black Females	47	61	140	186	144	115
Black Males	68	110	176	159	85	48
White Females	120	137	190	190	123	86
White Males	79	140	266	224	97	52
